# A Shared Food Source Is Not Necessary to Elicit Inequity Aversion in Dogs

**DOI:** 10.3389/fpsyg.2019.00413

**Published:** 2019-03-13

**Authors:** Jim McGetrick, Sabrina Ausserwöger, Ingrid Leidinger, Claudia Attar, Friederike Range

**Affiliations:** ^1^Domestication Lab, Konrad Lorenz Institute of Ethology, University of Veterinary Medicine, Vienna, Austria; ^2^Comparative Cognition Unit, Messerli Research Institute, University of Veterinary Medicine, Medical University of Vienna & University of Vienna, Vienna, Austria

**Keywords:** dog, inequity aversion, cooperation, competition, social comparison

## Abstract

Cooperative interactions frequently result in the acquisition of resources that have to be shared. Distribution of such resources should be equitable for cooperation to be beneficial. One mechanism thought to maintain cooperation through promotion of equitable reward distribution is inequity aversion, the resistance to inequitable outcomes. Inequity aversion has been demonstrated in many non-human animal species. It is not yet clear whether inequity aversion is limited to situations in which resources are shared; however, a recent study on inequity aversion in dogs, in which reward sources were separated, failed to elicit inequity aversion, hinting at the possible necessity of a shared resource for eliciting inequity aversion. Here, we employed a modified version of the previously used paw task to test the hypothesis that a shared food source is necessary to elicit inequity aversion in dogs. In our study, an experimenter asked pairs of dogs for their paw and rewarded them equally or unequally; however, unlike the standard paw task, the rewards for each dog came from separate food bowls. Dogs displayed the typical basic aversion to inequity despite the lack of a shared food source. These results suggest that a shared food source is not necessary to elicit inequity aversion and that separation of food sources does not explain the recent failure to elicit inequity aversion in dogs. Our findings may also be reflective of the variety of situations in which inequity aversion is potentially applied, the mechanisms underlying inequity aversion in dogs, and the behavioural contexts from which inequity aversion initially evolved.

## Introduction

Cooperative interactions among animals frequently result in the acquisition of shared resources. For example, a cooperative hunt among chimpanzees (*Pan troglodytes*), wolves (*Canis lupus*), or lions (*Panthera leo*) can result in the acquisition of a single carcass (Mech, 1970; Scheel and Packer, 1991; Boesch, 1994, 2002; MacNulty et al., 2014; Gilby et al., 2015). How such shared resources are distributed is not trivial, as individuals can vary in the effort they invest (Scheel and Packer, 1991), the specific roles they play in the hunt (Stander, 1992; Boesch, 2002), and the importance of the contributions they make (Gilby et al., 2015), in acquiring the resource. Furthermore, competition arises over such valuable and limited resources (Boesch and Boesch, 1989) and cheating can occur, whereby individuals contribute little but still attempt to gain access to the resource, or whereby individuals exploit a resource through monopolisation (Strum, 1981; Packer and Ruttan, 1988; Scheel and Packer, 1991; Boesch, 1994; Gilby et al., 2017; Samuni et al., 2018). Distribution of the resource should, theoretically, benefit cooperators over cheaters in order for stable cooperation to evolve (Boesch, 1994), and recent evidence from observations of hunting among chimpanzees indicates that hunt participation does in fact predict meat distribution despite similar begging duration and begging intensity between hunt participants and non-participant bystanders (i.e. cheaters) (Samuni et al., 2018).

One mechanism thought to contribute to the maintenance of cooperation among unrelated individuals, through promotion of equitable sharing, is inequity aversion (Fehr and Schmidt, 1999; Brosnan, 2011; Brosnan and Bshary, 2016). Inequity aversion can be defined as resistance to inequitable outcomes (Fehr and Schmidt, 1999), implying that inequity averse individuals will reject rewards or will refuse to work under conditions of inequity. Such inequity averse behaviour has been demonstrated in a wide variety of animal species, primarily using a token-exchange task in which two conspecifics give tokens to an experimenter and receive rewards of varying quality in return (Brosnan and de Waal, 2003; Brosnan et al., 2005) (for review, and examples of inequity averse species, see Brosnan and de Waal, 2014, and McGetrick and Range, 2018). Initial reports of inequity aversion in primates were controversial due to potential alternative explanations such as food expectation or negative contrast (i.e. reduced performance due to a downshift in reward quality) (Wynne, 2004; Roma et al., 2006; Neiworth et al., 2009); however, these alternative explanations were ruled out in later studies (van Wolkenten et al., 2007; Brosnan et al., 2010; Hopper et al., 2014; see also McGetrick and Range, 2018, for a discussion of the alternative explanations).

Given that inequity aversion putatively evolved to maintain cooperation (Brosnan, 2011) and prevent exploitation of shared resources, inequity aversion may only be functionally relevant to situations in which a limited resource can be shared or monopolised. In such cases, it is in the interest of each individual to prevent disproportionate depletion of the shared resource by peers. However, if two individuals' payoffs differ in quantity or quality, but these payoffs are obtained from separate food sources, or from independent foraging events, concerns relating to inequity may be less important; a peer's greater success in such situations does not limit one's own potential to obtain payoffs. Neiworth et al. (2009), similarly, posited that social comparisons (i.e. comparing one's own payoffs with those of a partner) may be more important in situations in which competition exists due to limited availability of resources.

The majority of inequity aversion exchange tasks that have been carried out to date involve the storage of food rewards in one or two shared containers, in front of experimental participants (Brosnan and de Waal, 2003; Brosnan et al., 2005; Fontenot et al., 2007; van Wolkenten et al., 2007; Bräuer et al., 2009; Wascher and Bugnyar, 2013; Heaney et al., 2017). Thus, it is not clear whether negative responses to unequal distributions in non-human animals are limited to situations in which rewards are distributed from a shared pool.

Some evidence for separation of food sources influencing responses in cooperative contexts comes from a cooperative “bar-pull” task with capuchin monkeys, carried out by de Waal and Davis (2003). Pairs of unrelated adult capuchin monkeys were less likely to successfully cooperate if the available rewards were clumped than if they were dispersed (i.e. with separate rewards for each individual). In this study, dominant individuals obtained the most food rewards under the clumped condition; thus, the results suggest that the lower tendency to cooperate when rewards were clumped, was due to anticipation of competition and inequity. Although inequity was shown to influence cooperation among capuchin monkeys in the bar-pull task when rewards (of different quality) were separated (see Brosnan et al., 2006), de Waal and Davis' (2003) results provide evidence that individuals are attentive to whether rewards are monopolisable and that this can influence subsequent decisions in cooperative contexts.

Domestic dogs provide a useful model species for the study of inequity aversion in non-human animals. Dogs were the first non-primate species shown to respond negatively to inequity and are now one of the best-studied species in the field. In the initial study, Range et al. (2009) asked pairs of dogs to give their paw, alternately, in return for rewards of either the same or different quality, or for no rewards at all. In contrast to many primate species tested to date, dogs did not respond to inequity relating to reward quality, but when subjects were unrewarded in the presence of a rewarded conspecific, they refused to continue giving their paw. Subjects did, however, comply with the experimenter's commands when unrewarded but alone, ruling out the possibility that the dogs' responses were due simply to the lack of reward rather than inequity. Furthermore, food expectation and negative contrast were ruled out as all rewards were visible to subjects across all conditions, and subjects' rewards were downshifted equally in the inequity and control condition. Overall, these results suggest that dogs possess a basic form of inequity aversion and this response appears to be robust, as it has since been replicated in another “paw task” study (Brucks et al., 2016).

Over the past decade, research on domestic dogs has attempted to elucidate factors that influence inequity aversion, in animals (Range et al., 2012; Brucks et al., 2017b). Interestingly, a recent attempt to investigate the influence of the human experimenter, using a novel task but similar paradigm, failed to find inequity aversion in dogs (Brucks et al., 2017a). In this study, a subject and a partner dog were situated in adjacent enclosures, but rather than having to give their paw to an experimenter, they were required to press a buzzer once it was pushed into their enclosure. Each time a dog pressed the buzzer, a food reward was pushed into its enclosure (or not, depending on the condition), on top of a small box. In the “experimenter present” version of this task, an experimenter sat in front of the two dogs and issued commands to press the buzzers, while they also pushed the boxes with rewards into the enclosures. In the “experimenter absent” version, no experimenter was visible to the dogs and no commands were issued. In both versions of these tasks, two experimenters were hidden behind a curtain and were responsible for controlling the buzzers, and for baiting the boxes with rewards. Dogs did not exhibit inequity aversion in either version of this task.

The factors responsible for a lack of inequity aversion in Brucks et al.'s (2017a) study are not yet clear. However, it is a particularly intriguing finding for multiple reasons. First, the task was paradigmatically similar to the paw task, which consistently elicits inequity aversion in dogs (Range et al., 2009; Brucks et al., 2016). Second, many of the subjects used in this “buzzer task” were also tested in the paw task in which they exhibited inequity aversion. Third, and perhaps most importantly, the buzzer task was recently used successfully by Essler et al. (2017) to demonstrate inequity aversion in wolves and pack-living dogs.

This discrepancy in the research on inequity aversion in dogs may be informative in the context of understanding the effects of shared resources. Although a variety of factors differed across these tasks (e.g. rearing and husbandry conditions of the subjects, the presence of an owner during testing, testing indoor vs. outdoor), a particularly salient feature of those tasks that elicited inequity aversion, is that all food rewards were distributed from a single, shared food bowl in front of, and equidistant from, both dogs (Range et al., 2009; Brucks et al., 2016; Essler et al., 2017). In contrast, in the buzzer task carried out with pet dogs by Brucks et al. (2017a), which failed to elicit inequity aversion, food rewards were separated: pieces of food were positioned on containers directly in front of each dog such that they each had their own food source.

In an attempt to contribute to a better understanding of factors that influence inequity aversion, and to explain the lack of inequity aversion observed by Brucks et al. (2017a), we tested the hypothesis that a shared food source is necessary to elicit inequity aversion in dogs, by employing a modified version of the paw task: rather than using a shared food bowl from which rewards were distributed, the subject and partner dog each had its own bowl from which it received its rewards.

## Materials and Methods

Ethical approval was obtained from the ethics commission (Ethik- und Tierschutzkommission [ETK]) of the University of Veterinary Medicine Vienna (ethical approval: ETK - 06/11/2016). Additionally, owners were required to sign a consent form prior to participation.

### Subjects

Twelve dog dyads (i.e. 24 subjects; 12 males, 12 females) of various breeds, including mixed breeds, were recruited for this study (see Table 1 for details). In order to be included in the study, dogs in each dyad were required to already be able to give the paw and to sit on command. Additionally, a dyad was only included in the study if they had been living in the same household for longer than 1 year and had not participated in any previous inequity aversion study. Furthermore, dogs were only included if they did not show any food aggression. In order to assess each subject's ability to give its paw to the experimenter on command, on the first test day each subject was asked for its paw 15 times in a row. The dogs received a reward the first 5 times, the next 5 times they were unrewarded, while the last 5 times they were rewarded again. All tests were conducted between October 2016 and January 2017 in a test room (7 × 6 m) at the Clever Dog Laboratory, located at the University of Veterinary Medicine Vienna. Three female experimenters conducted the experiments; however, each of these experimenters took responsibility for specific dyads, carrying out all conditions with these dogs. One dyad was excluded due to low motivation to perform the task; consequently 22 subjects were included in the final analysis.

**Table 1:** List of participants in the study, including information regarding dyad number, sex, breed, age, and rewards used.

**Dyad**	**Name**	**Sex**	**Breed**	**Age (yrs.)**	**LVR**	**HVR**
1	Sammy	f	Jack Russel Terrier	11.0	Semi-moist food	Cheese
1	Jazz	m	German Shepherd	5.3	Semi-moist food	Cheese
2	Luise	f	Mix	2.8	Sausage	Semi-moist food
2	Odin	m	German Shepherd	2.8	Sausage	Semi-moist food
3	Balian	m	Border Collie	7.0	Dry food	Sausage
3	Carlisle	m	Border Collie	6.2	Dry food	Sausage
4	Diesel	m	Australian Shepherd	4.8	Dry food	Cheese
4	Eyko	m	Australian Shepherd	7.8	Dry food	Cheese
5	Chuck	m	Australian Shepherd	6.8	Dry food	Cheese
5	Ruby	f	Australian Shepherd	5.0	Dry food	Cheese
6	Lara	f	Mix	6.8	Dry food	Treat-sticks
6	Amy	f	Labrador Retriever	4.6	Dry food	Treat-sticks
7	Cliff	m	Smooth Collie	7.7	Dry food	Sausage
7	Soferl	f	Mix	9.3	Dry food	Sausage
8	Barolo	m	Standard Poodle	6.9	Dry food	Sausage
8	Raico	m	Standard Poodle	6.4	Dry food	Sausage
9	Bambi	f	Mix	4.0	Dry food	Cheese
9	Ginger	f	Mix	13.2	Dry food	Cheese
10	Drake	m	Smooth Collie	6.9	Dry food	Cheese
10	Quainty	f	Smooth Collie	5.0	Dry food	Cheese
11	Smoky	f	Shetland Sheepdog	3.9	Kibble	Treat-sticks
11	Vanilla	f	Bearded Collie	12.3	Kibble	Treat-sticks
12	Mateja^*^	f	Mix	8.3	Dry food	Semi-moist food
12	Furio^*^	m	Whippet	3.2	Dry food	Semi-moist food

* *These subjects were excluded from the final analysis due to low motivation to perform the task throughout the experiment. LVR, low value reward; HVR, high value reward*.

### Food Preference Test

In order to assign a low value reward (LVR) and high value reward (HVR) to each dyad, a food preference test was carried out. Two candidate reward types were initially selected based on the owner's subjective assessment. The LVR had to be a food type that that the dogs were sufficiently motivated to work for, whereas the HVR was a food type that they preferred to the LVR. The LVR and HVR were kept the same for both dogs in a dyad, though the food preference test was carried out separately for both dogs to confirm that their preference was the same. In order for a food type to be considered suitable for the experiment, it had to be relatively dry so that it could be handled easily during the experiment, while it also had to be divisible into equally sized pieces.

During the food preference test, the owner was required to sit on a chair with their dog held on the leash. The experimenter baited two green plastic lids, one for each food type, while in view of the dog, at a distance of ~1.2 m from it. The experimenter subsequently leaned forward, holding both lids ~20 cm from the dog's nose allowing the dog to sniff both food types. The experimenter then leaned back and placed the lids on the ground, 50 cm apart and equidistant from the dog (~60–70 cm). The owner released their dog once the experimenter placed their hands on their lap and looked to the ground, allowing the dog to make its choice. Once the dog had made its choice, the experimenter removed the second lid, thereby preventing the dog from gaining access to the second reward. The owner was requested to look straight ahead to the wall behind the experimenter during the test so as to reduce their potential influence on the dog's choice.

This test was carried out for 12 trials with the relative positions of the two reward types being switched after each trial. The relative position of the HVR on the first trial was counterbalanced across dogs. A particular food type was considered the preferred food when it was chosen on at least 9 out of 12 trials (binomial *p* < 0.02). If no clear food preference was determined within the first session, the session was repeated, a maximum of 3 times until a food preference was reached. If there was no clear preference after 3 sessions, new food types were chosen and the process was repeated. The final LVRs and HVRs for each dyad are listed in Table 1.

### Paw Task

**Figure 1:**
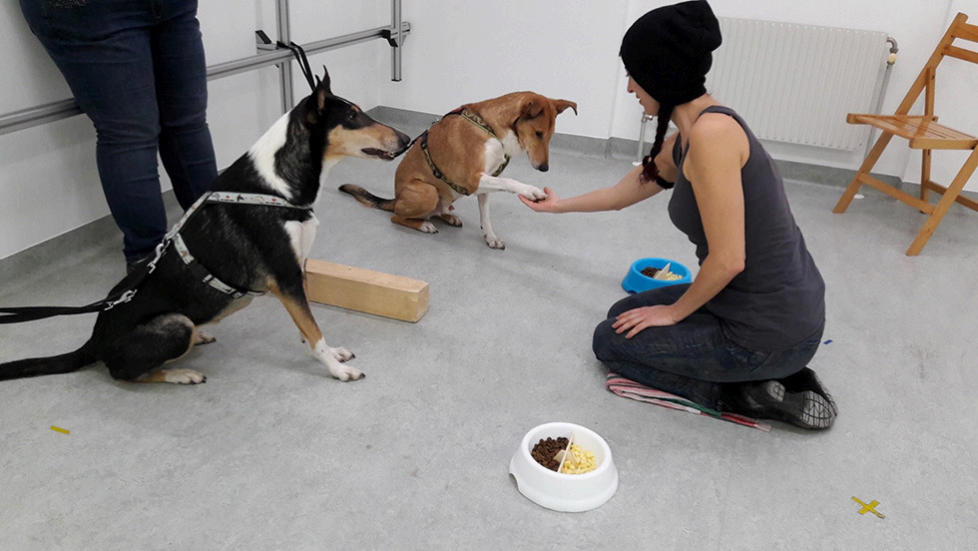
Paw task setup. Written, informed consent was obtained for the publication of this image from the photographed individual.

The paw task carried out in this study matched the procedure used by Brucks et al. (2016), except where otherwise stated. The most important modification to the procedure was the use of a separate bowl for each dog in the dyad, rather than a shared bowl (see Figure 1 for setup).

The two dogs were positioned next to each other, separated by a wooden block (60 × 10 × 10 cm), which was placed on the ground. The dogs were both kept on leashes of equal length that were tied to separate metal rods ~2 m apart, while the owner stood passively in between with their back to the wall. The experimenter knelt in front of the dogs, at such a distance that they could comfortably ask for the paw while also being able to reach for food rewards from the bowls, and hand the food to the dogs.

The two food bowls were used in all conditions of this experiment. The food bowls each had a diameter of 30 cm and contained both the LVR and HVR, separated by a thin piece of plywood. Each bowl was positioned directly in front of, and ~50 cm from, one of the dogs. Thus, there was a bowl of food in front of each dog. The pieces of LVR were always in the portion of the bowl closest to the dog, and both reward types were visible to the dogs. One bowl was white, while the other was sky blue; each dog stuck with a particular bowl colour throughout the study.

The experiment began once the experimenter knelt in front of the dogs. Beginning with the partner (i.e. the individual not being subjected to disadvantageous inequity [see Table 2]), the experimenter asked the dogs alternately to give their paw, using the German command “Pfote!” and presenting the palm of the hand to the dog. Each dog was asked for its paw a total of 30 times in a session; thus, a session lasted for 60 trials with 30 trials for each dog unless the session was terminated due to refusal by the subject to participate. Each time the subject or partner gave its paw successfully, the experimenter rewarded it (or not, depending on the condition), with food from the bowl in front of that particular dog. The rewarding procedure itself differed from previous paw task studies (Range et al., 2009; Brucks et al., 2016) in terms of how the food was moved. Brucks et al. (2016) and Range et al. (2009) lifted the food reward up in between the two dogs and held it there momentarily (in order to ensure quality perception) before handing it to the actor. In this study, the reward was not moved into the centre but was lifted up over the respective bowl to strengthen the separation of the two food sources. Additionally, the experimenter used their left hand for commands and food rewards for dogs on their left and used their right hand for commands and food rewards for dogs on their right; in previous studies (Range et al., 2009; Brucks et al., 2016) one hand was used for giving commands and the other for handing over the reward, or the same hand was used for all commands and rewards.

**Table 2:** Test conditions, including rewards given to the subject and partner in each condition.

**Social conditions**	**Subject**	**Partner**
Equity test (ET)	LVR	LVR
Quality inequity (QI)	LVR	HVR
Reward inequity (RI)	—	HVR
Food control (FC)	HVR lifted, LVR given	HVR lifted, LVR given
**Asocial conditions**	**Subject**	**No partner**
Assessment control (AC)	LVR	—^*^
No-Reward control (NR)^‡^	—	—^*^

* *In these conditions, although no partner was present, after each trial with the subject, a piece of food was lifted from the partner's bowl, and moved to the partner's position as though the experimenter was providing food to a dog. This piece of food was immediately returned to the bowl. A piece of LVR was moved to the partner's position in the AC condition (after the subject received its reward), whereas a piece of HVR was moved to the partner's position in the NR condition.^‡^In Range et al. (2009) and Brucks et al. (2016) a piece of LVR rather than HVR was moved to the partner's position in the NR condition*.

If the subject refused to follow the paw command (i.e. if it did not give the paw within ~1 s of the experimenter issuing the command) on a particular trial, the experimenter asked the subject for its paw a total of 11 times within that trial, with ~1 s in between each command and with the dog's name being called after the 6th command. The session was terminated if subjects refused to give their paw after these commands.

Dogs were required to sit before they were asked for their paw. If they were not already sitting, the experimenter asked them to sit using an outstretched index finger and the German command “Sitz!”. A correct response to the sit command was not rewarded. If dogs did not obey this initial command, it was repeated up to 10 times with an ~1 s pause between each command, and with the experimenter calling the dog's name after the 5th command. If the subject did not sit after repetition of the sit command 10 times, the experimenter turned their attention to the partner, and rewarded it for an extra 5 paw trials (which were not included as part of the 60 trials making up the session), before returning to the subject and repeating the sit commands. A similar criterion was applied to the asocial conditions: the food was moved for 5 extra trials to the partner's empty position. The extra paw trials are standard in the paw task procedure (Range et al., 2009; Brucks et al., 2016) and serve to prevent a fake refusal due to the lack of reward for a correct response to a sit command. If the subject changed position dramatically during these repetitions (e.g. changing from a lying to a standing position), the experimenter started the 10 commands again. Failure by the subject to sit after 10 sit commands led to termination of the session.

In contrast with Range et al. (2009), but in keeping with the method of Brucks et al. (2016), warm-up trials were not conducted before test conditions, nor were dogs handed pieces of food before the test conditions.

A second experimenter, sitting ~2 m away from the dogs, kept track of the trial number and the number of sit and paw commands issued within each trial, while they also recorded the results on a data sheet and informed the first experimenter when the appropriate number of trials or sit and paw commands had been completed.

### Test Conditions

Subjects experienced six experimental conditions, in accordance with the methods of Brucks et al. (2016) (see Table 2). These included four social conditions with both dogs present and acting either as the partner or the subject: the Equity Test (ET) (or baseline) condition (both dogs are rewarded with LVR), the Food Control (FC) condition (both dogs are rewarded with LVR, but HVR is lifted up and put back first in order to induce frustration), the Quality Inequity (QI) condition (the partner is rewarded with HVR, whereas the subject is rewarded with LVR), and the Reward Inequity (RI) condition (the partner is rewarded with HVR, whereas the subject is not rewarded; in Range et al. (2009), the partner was rewarded with LVR). Additionally, there were two asocial conditions, in which the subject was tested alone and no partner dog was present in the room: the Assessment Control (AC) condition (the subject is rewarded with LVR) and the No-Reward (NR) condition (the subject is unrewarded to control for the possibility that any refusals in the RI condition are simply due to the absence of reward). In the asocial conditions, after each trial completed by the subject, a piece of LVR (AC condition) or HVR (NR condition) was lifted up, moved to the partner's empty position and then put back into the bowl in order to control for the movement of the food that occurs in the RI condition when feeding the partner (Range et al., 2009, and Brucks et al., 2016, moved LVR in both asocial conditions). Two conditions were carried out per day with a 10-min break in between, which is similar to Brucks et al. (2016), who included a food tolerance test and 10-min break in between, and to Range et al. (2009), who included a 15-min break. During this break the two dogs were free to roam around the test room following removal of the food bowls. The asocial conditions (the AC and NR conditions) were always carried out on the same day with the AC condition first; furthermore, the two conditions in which the subject did not receive a reward (the RI and NR conditions), were never carried out as the first condition on the first test day. Otherwise, the conditions were carried out in a random order. After one of the dogs in the dyad had experienced all conditions as the subject, the roles were reversed and the partner then experienced all conditions as the subject.

### Coding and Statistical Analyses

All test conditions were recorded on video and then the commands and behaviours were coded using Solomon Coder beta 17.03.22 (copyright 2017 by András Péter; https://solomoncoder.com/). The number of trials in which the subject gave the paw and the number of paw and sit commands were coded. The number of stress signals (lip-licking, yawning, shaking, and scratching) issued by the dog was also coded. Additionally, the duration of gazing (based on orientation of the head) at the partner, the partner's bowl, and the subject's own bowl were coded. Each video was coded by the individual who performed the experiment with that particular dyad. Video footage for six test sessions was unavailable (AC condition × 5; ET condition × 1); thus, no coding was carried out for these sessions. For cases in which video footage was unavailable, the number of times the subject gave the paw, and the number of commands issued, was taken from the score sheet recorded by the second experimenter. In an additional two videos (ET condition × 1; RI condition × 1) stress behaviours were not coded due to poor visibility of the subject. Statistical analyses were carried out using R (version 3.4.2; R Core Team, 2017), primarily using the package “lme4” (Bates et al., 2015), and plots were created in R using the package “ggplot2” (Wickham, 2009).

The number of trials completed (i.e. the number of times the subject gave the paw) was analysed as a proportion of the number of planned trials (i.e. 30) using a binomial Generalised Linear Mixed Model (GLMM). The fixed effects included in the model were condition, test day (structured as a factor with three levels i.e. first, second, third), whether the condition was tested first or second on a particular day, and whether the individual began the study as a subject or partner. The latter three fixed effects were included to rule out order effects and carry-over effects. The random effects included in the model were observation ID, and subject ID nested within dyad ID.

Due to overdispersion in poisson models, the number of commands issued (paw commands and sit commands combined) was analysed using a GLMM with penalized quasi-likelihood (quasi-poisson family), in the “MASS” package (Venables and Ripley, 2002), and the number of stress behaviours exhibited by the subjects was analysed using a negative binomial GLMM, in the package “lme4”. The fixed effects included in both models were identical to those above. In both models, subject ID nested within dyad ID was included as a random effect. In the analysis of the number of commands issued the log of the number of trials completed was included as an offset term; thus, the number of commands issued in a test was corrected for the number of trials the subjects completed. One observation was excluded from the analysis of the number of commands issued per trial, as the subject did not give the paw in that condition, precluding the use of that data point in the offset term. The log of test duration was included as an offset term in the model analysing the number of stress behaviours; thus, the number of stress behaviours displayed was corrected for the test duration.

Durations (i.e. duration of gazing at the partner, the partner's bowl, and the subject's own bowl) were analysed as a proportion of the total test duration using a binomial GLMM. The same fixed effects as above were included in the models and observation ID, as well as subject ID nested within dyad ID, were included as random effects. Only social conditions were included in the analysis of duration of gazing at the partner, and only one test session was missing for this variable due to unavailability of video footage.

The duration of gaze at the partner's compared with the subject's own bowl in the ET (baseline) condition was analysed using a Linear Mixed Effects Model (LMEM) with the same fixed effects as above and with subject ID nested within dyad ID as the random effect. Normality was assessed using a Shapiro-Wilk normality test and homoscedasticity was assessed using a Breusch-Pagan test in the package “lmtest” (Zeileis and Hothorn, 2002); visual inspection of diagnostic plots including histograms, qqplots, and residual vs. fitted plots, was also used to assess normality and homoscedasticity. The initial model did not fit the assumption of normally distributed residuals; therefore, the Box-Cox transformation method was applied, using the package “MASS”, and the appropriate transformation was applied to the response variable to achieve normally distributed residuals (Venables and Ripley, 2002).

In all cases (except for the quasipoisson GLMM), a full model (i.e. a model including the fixed effect of interest, i.e. condition) was compared with a null model (i.e. the same model with the fixed effect of interest removed) using a likelihood ratio test (using the R function “anova” and setting the “test” argument to “Chisq”) to determine whether the fixed effect of interest had a significant effect. P-values for pairwise comparisons in models constructed using the package “lme4” were obtained using the package “lmerTest” (Kuznetsova et al., 2017).

A fourth experimenter coded 20% of the videos, coding an approximately equal number of videos from each of the three experimenters. Inter-observer reliability was analysed using the intra-class correlation coefficient from the package “irr” (Gamer et al., 2012) (Intra-class correlation coefficient [ICC, consistency]: number of times the paw was given on command: ICC (two-way, consistency) = 0.998, *p* < 0.001; number of paw commands: ICC (two-way, consistency) = 0.999, *p* < 0.001; number of stress signals: ICC (two-way, consistency) = 0.892, *p* < 0.001; duration of gaze at the partner: ICC (two-way, consistency) = 0.867, *p* < 0.001; duration of gaze at the partner's bowl: ICC (two-way, consistency) = 0.866, *p* < 0.001; duration of gaze at own bowl: ICC (two-way, consistency) = 0.952, *p* < 0.001).

## Results

### Trials Completed

**Figure 2:**
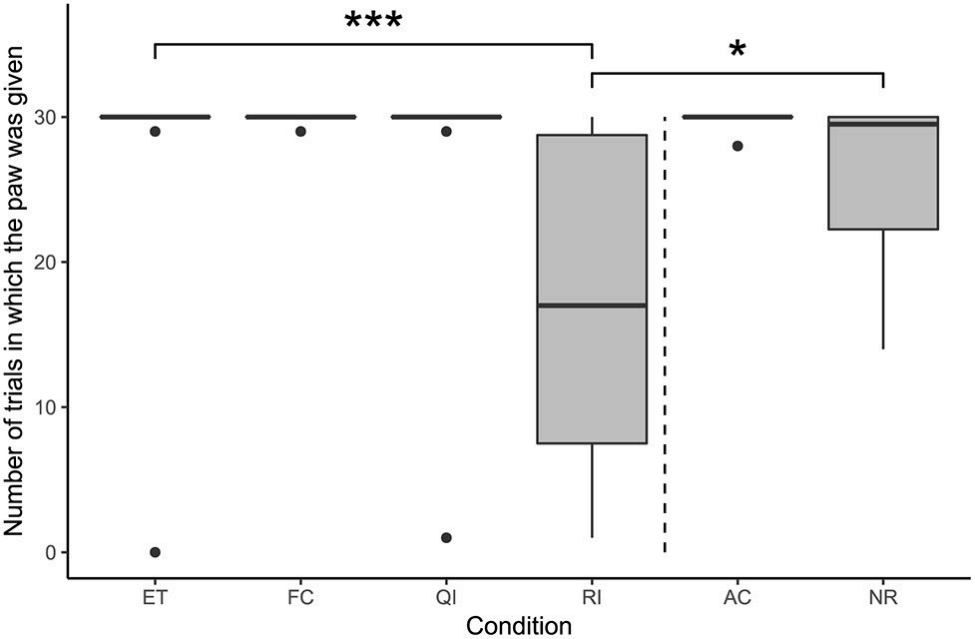
Number of times the paw was given on command in each condition of the paw task (N = 22). Boxes show interquartile range, black bar represents the median, whiskers represent the range of data within 1.5 times the interquartile range from the upper and lower hinge, black dots represent outliers, and dashed, vertical line separates social and asocial conditions. Asterisks indicate significant differences between conditions; relevant comparisons between social conditions and between unrewarded conditions are presented (*p < 0.05, ***p < 0.001).

Condition had a significant effect on the number of trials in which the subjects gave the paw, according to the comparison between the full model (i.e. the model with the fixed effect of condition included) and the null model (i.e. the model with the fixed effect of condition excluded; likelihood ratio test: χ^2^ = 28.95, df = 5, *p* < 0.001; see Figure 2). The number of times the subjects gave the paw was significantly lower in the RI condition compared with the ET condition (GLMM: β = −6.48, S.E. = 1.46, *p* < 0.001). Additionally, the number of times the paw was given in the RI condition was also significantly lower than that in the NR condition (GLMM: β = −3.62, S.E. = 1.44, *p* = 0.011). The remaining social conditions did not differ significantly from the ET condition (GLMM: FC vs. ET: β = 2.21, S.E. = 2.08, *p* = 0.290; QI vs. ET: β = 0.91, S.E. = 1.65, *p* = 0.581).

### Commands

The number of combined paw commands and sit commands issued per trial was significantly larger in the RI condition compared with that in the ET condition and the NR condition (GLMM: RI vs. ET: β = 0.69, S.E. = 0.31, *p* < 0.001; RI vs. NR: β = 0.39, S.E. = 0.12, *p* = 0.002). The response for the two remaining social conditions did not differ significantly from that for the ET condition (GLMM: FC vs. ET: β = −0.02, S.E. = 0.13, *p* = 0.905; QI vs. ET: β = 0.08, S.E. = 0.13, *p* = 0.55).

### Stress

Condition did not have a significant effect on the number of stress signals displayed (corrected for test duration) according to the full-null model comparison (likelihood ratio test: χ^2^ = 6.82, df = 5, *p* = 0.235). Therefore, the full model was not analysed further.

### Gazing

#### Gazing at the Partner

**Figure 3:**
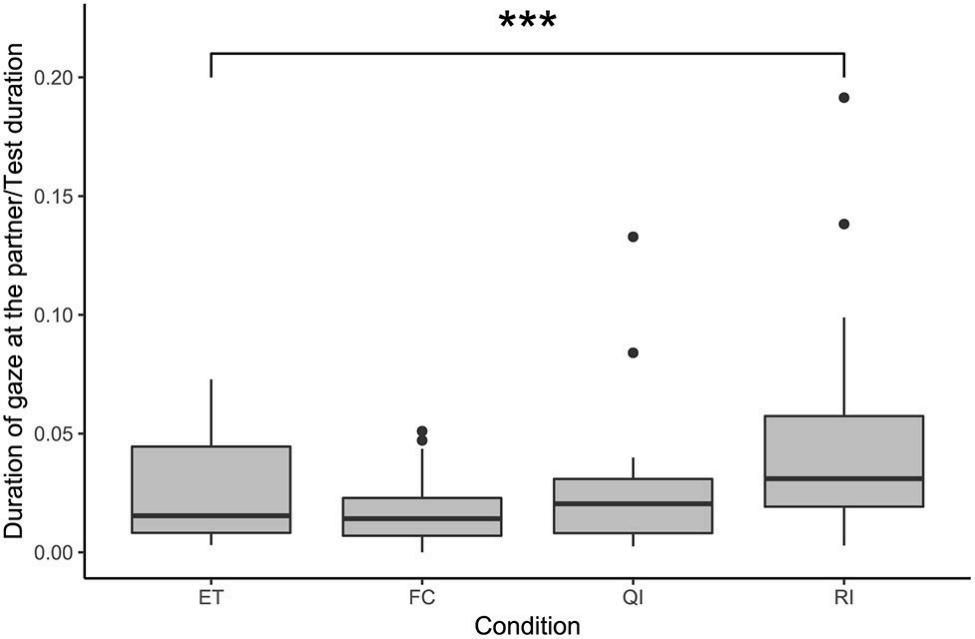
Duration of gaze at the partner, as a proportion of test duration, in the social conditions. Boxes show interquartile range, black bar represents the median, whiskers represent the range of data within 1.5 times the interquartile range from the upper and lower hinge, and black dots represent outliers. Asterisks indicate significant differences between conditions; relevant comparisons between social conditions are presented (***p < 0.001).

Condition had a significant effect on the duration of gaze at the partner according to the comparison between the full model and the null model (likelihood ratio test: χ^2^ = 2.83, df = 3, *p* < 0.001; see Figure 3). Subjects gazed for significantly longer at the partner in the RI condition compared with the ET condition (GLMM: RI vs. ET: β = 0.86, S.E. = 0.21, *p* < 0.001). Duration of gazing at the partner did not differ from the ET condition in the two other social conditions (GLMM: FC vs. ET: β = −0.10, S.E. = 0.22, *p* = 0.633; QI vs. ET: β = 0.22, S.E. = 0.21, *p* = 0.305). We also observed, *post-hoc*, an effect of whether a test was conducted first or second on a test day, on the duration of gazing at the partner, with significantly less gazing if a test was second (GLMM: β = −0.41, S.E. = 0.15, *p* = 0.006).

#### Gazing at the Partner's Bowl

**Figure 4:**
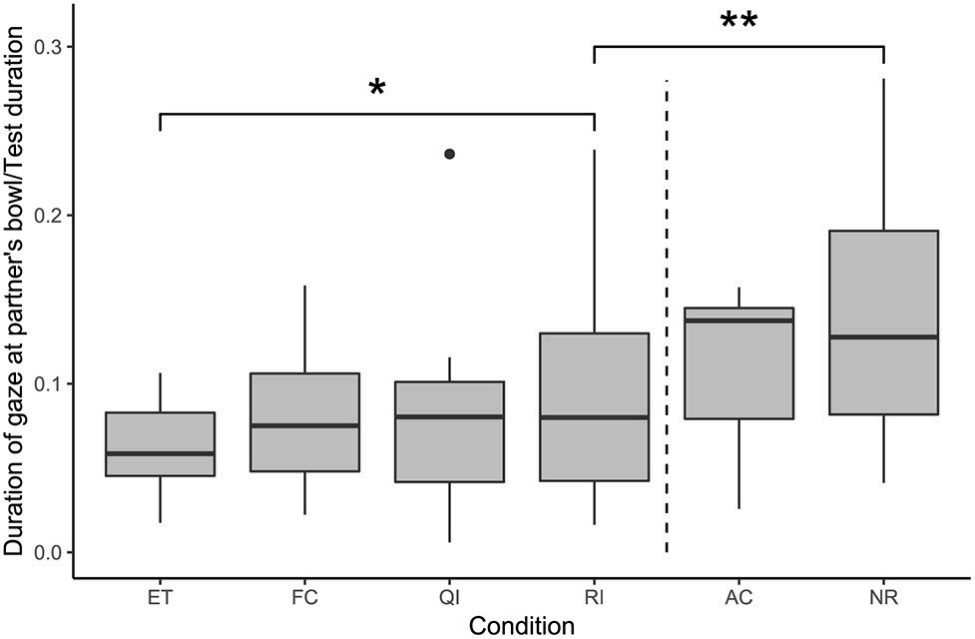
Duration of gaze at the partner's bowl, as a proportion of test duration, in each condition. Boxes show interquartile range, black bar represents the median, whiskers represent the range of data within 1.5 times the interquartile range from the upper and lower hinge, black dots represent outliers, and dashed, vertical line separates social and asocial conditions. Asterisks indicate significant differences between conditions; relevant comparisons between social conditions and between unrewarded conditions are presented (*p < 0.05, **p < 0.01).

The results of a full-null model comparison indicated a significant effect of condition on the duration of gazing at the partner's bowl (likelihood ratio test: χ^2^ = 32.83, df = 5, *p* < 0.001; see Figure 4). Subjects spent significantly more time gazing at the partner's bowl in the RI condition than in the ET condition, while the FC and QI conditions did not differ from the ET condition in this regard (GLMM: RI vs. ET: β = 0.35, S.E. = 0.17, *p* = 0.034; FC vs. ET: β = 0.22, S.E. = 0.16, *p* = 0.170; QI vs. ET: β = 0.19, S.E. = 0.16, *p* = 0.246). Subjects spent significantly less time gazing at the partner's bowl in the RI condition than in the NR condition (GLMM: RI vs. NR: β = −0.51, S.E. = 0.16, *p* = 0.002).

#### Gazing at the Subject's Own Bowl

**Figure 5:**
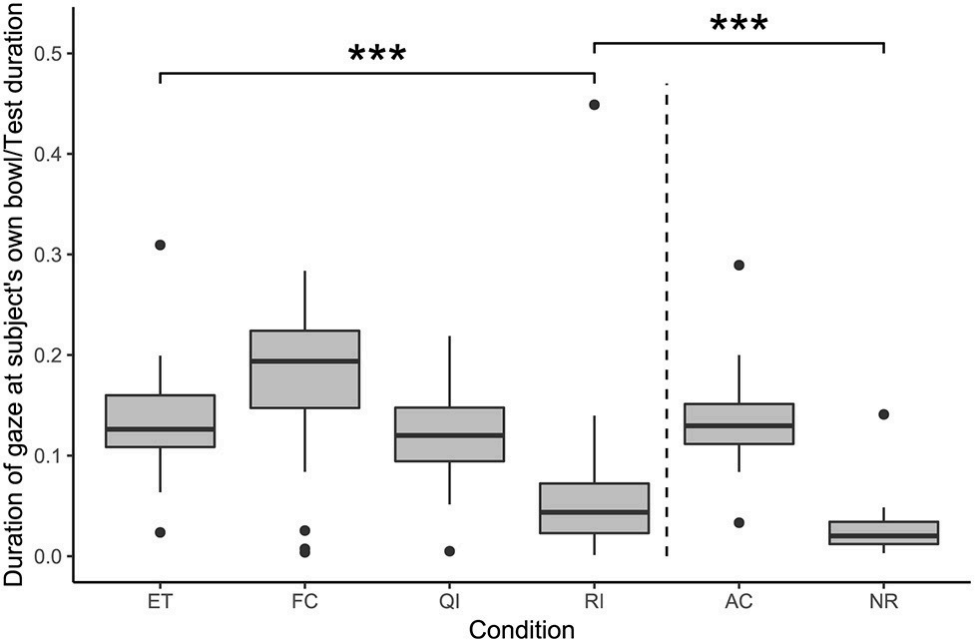
Duration of gaze at the subject's own bowl, as a proportion of test duration, in each condition. Boxes show interquartile range, black bar represents the median, whiskers represent the range of data within 1.5 times the interquartile range from the upper and lower hinge, black dots represent outliers, and dashed, vertical line separates social and asocial conditions. Asterisks indicate significant differences between conditions; relevant comparisons between social conditions and between unrewarded conditions are presented (***p < 0.001).

A full-null model comparison revealed that condition also had a significant effect on the duration of gazing at the subject's own bowl (likelihood ratio test: χ^2^ = 80.85, df = 5, *p* < 0.001; see Figure 5). Subjects spent significantly less time gazing at their own bowl in the RI condition compared with in the ET condition (GLMM: RI vs. ET: β = −1.09, S.E. = 0.22, *p* < 0.001). The remaining social conditions did not differ from the ET condition in this regard (GLMM: FC vs. ET: β = 0.15, S.E. = 0.21, *p* = 0.480; QI vs. ET: β = −0.09, S.E. = 0.21, *p* = 0.675). Additionally, subjects spent significantly more time gazing at their own bowl in the RI condition than in the NR condition (GLMM: RI vs. NR: β = 0.86, S.E. = 0.24, *p* < 0.001).

#### Gazing at the Partner's Bowl vs. the Subject's Own Bowl in the ET Condition

Subjects spent significantly longer gazing at their own bowl than at the partner's bowl in the ET (baseline) condition (likelihood ratio test: χ^2^ = 32.97, df = 1, *p* < 0.001; LMEM: subject's own bowl vs. partner's bowl: β = 2.17, S.E. = 0.27, *p* < 0.001).

## Discussion

In this study we tested the hypothesis that a shared food source is necessary to elicit inequity aversion in dogs. To test this hypothesis, we carried out the paw task previously used to demonstrate inequity aversion in dogs (Range et al., 2009; Brucks et al., 2016); however, rather than using a shared food bowl from which food rewards were distributed, we separated the food source, assigning a bowl of food to each dog.

Despite the absence of a shared food source, subjects did exhibit the typical basic aversion to inequity, giving their paw significantly fewer times in the RI condition than in the ET and NR conditions. Furthermore, they required more paw and sit commands, per trial, in the RI compared with the ET and NR conditions. Subjects also gazed more at the partner in the RI condition than the ET condition. The dogs in this study did not respond negatively to inequity in reward quality (i.e. the QI condition), which is in keeping with previous paw task studies with dogs (Range et al., 2009; Brucks et al., 2016). Nevertheless, it does not seem that a shared food source is necessary to elicit dogs' typical negative response to inequity.

Several explanations might account for the lack of an effect of separation of the food source on inequity aversion in this study. First, the subjects in the study may not have actually perceived the food sources as separate in any meaningful sense. Although, elucidation of how individual animals perceive such situations is difficult, we found that the subjects spent longer gazing at their own bowl than at the partner's bowl in the ET (baseline) condition, suggesting that they did differentiate between the food sources to some extent, and that they perceived the food source on their side as being more important for them.

Second, although the food source itself was not shared, the experimenter responsible for distributing the food, and creating the inequitable outcome, was shared. The involvement of this shared experimenter could have influenced responses simply by directing subjects' attention to the payoff of the partner, thereby facilitating subjects' awareness of the inequity. Furthermore, the shared experimenter may play a crucial role as subjects may only respond negatively to inequity if they perceive another individual as being intentionally responsible for the inequity (i.e. social attribution), regardless of the physical origin of rewards (see Blount, 1995 for evidence of similar behaviour in human subjects).

Third, inequity aversion is thought to be important in reciprocal cooperation (Stevens and Hauser, 2004; Brosnan and Bshary, 2016), whereby resources or services are exchanged with a time delay in between (Trivers, 1971). In reciprocal cooperation, food items exchanged may be physically separated in time and space (Wilkinson, 1984; Rutte and Taborsky, 2008; Carter and Wilkinson, 2013); thus, inequity aversion and social comparison of payoffs would have to be applied to situations in which reward sources are physically separated. Dogs have, in fact, recently been shown to cooperate reciprocally (Gfrerer and Taborsky, 2017, 2018); therefore, physical separation of food sources may not be expected to have a major impact on dogs' assessments of inequity.

Fourth, there are numerous situations in which paying attention to the better payoffs of nearby conspecifics may be beneficial. For example, paying attention to the greater foraging success of others may provide opportunities to improve one's own foraging skills through social learning mechanisms such as imitation or emulation (Huber et al., 2009; Whiten, 2015; Whiten and van de Waal, 2018). Additionally, an individual that is attentive to the foraging success of a peer may afford itself the opportunity to steal such rewards or to coerce the peer into sharing (Kummer and Cords, 1991; de Waal, 2000; Galef et al., 2001; Stevens, 2004; Gilby, 2006; Morand-Ferron et al., 2006). In fact, theft of conspecifics' food may act as an additional social learning mechanism, providing information about what foods to eat (Galef and Whiskin, 1995; Galef et al., 2001).

Being attentive to the foraging success of conspecifics *and* having some emotional or behavioural reaction to the greater success of feeding conspecifics may also have benefits. These benefits may exist even if the conspecifics' rewards do not come from a directly contested food source. For example, as pointed out previously (Samuelson, 2004; Chen and Santos, 2006; Rayo and Becker, 2007), if one's peers are obtaining more food, it may be indicative of a greater availability of food at that particular time; such socially derived information may allow individuals to increase their foraging behaviour at the optimal time (Samuelson, 2004; Chen and Santos, 2006; Rayo and Becker, 2007). Social facilitation of feeding behaviour [i.e. stimulation of one's feeding behaviour by the feeding behaviour of social partners (Zajonc, 1965)] has, in fact, been observed in a variety of species (Harlow, 1932; Tolman, 1965; Sweeting et al., 1985; Glickman et al., 1999; Dindo et al., 2009; Herman, 2015), including dogs (Ross and Ross, 1949a,b). Importantly, the feeding behaviour of partners, in the RI condition of inequity tasks with dogs, may stimulate subjects' feeding behaviour or anticipation of food; however, in the absence of food receipt this may result in stress, culminating in subjects' discontinuation of the task. If social facilitation underlies dogs' negative response to inequity, we would not expect the physical location of the partner's rewards to have a major impact on this response, assuming the two animals are in close proximity to each other.

Finally, paying attention to peers' independently-obtained better payoffs, and experiencing an associated, negative emotional reaction, could facilitate the expression of punitive and spiteful behaviours towards such peers, which may be adaptive through fitness-levelling effects (Clutton-Brock and Parker, 1995; Jensen, 2010). In the paw task setting, a subject is limited in their ability to express punitive or spiteful behaviours; however, accruing negative emotions that would, in another context, drive punishment of a partner, may cause the subject to give up.

Interestingly, inequity aversion may have evolved from some of the potentially related contexts outlined here in which individuals pay attention and respond to the better success or payoffs of their peers (see Brosnan, 2006, and Chen and Santos, 2006, for further discussion on evolutionary precursors to inequity aversion). Additionally, if the latter examples of social facilitation, and punishment or spite, explain the responses of dogs in inequity tasks, then dogs' inequity aversion, as observed to date, may have little to do with cooperation, and may simply emerge as a side-effect of unrelated foraging and social behaviours.

Given that separation of the food source did not have an impact on dogs' response to inequity, the question of why dogs were not inequity averse in the buzzer task of Brucks et al. (2017a) remains. Another possible explanation, proposed by Brucks et al. (2017a), is that the task was not social enough to elicit inequity aversion, as there was no physical contact between the subject and an experimenter (even in the experimenter present version of the task). Essler et al. (2017) elicited inequity aversion in pack-living dogs using the buzzer task; therefore, the physical interaction of the dogs' paw on the experimenter's hand does not seem to be an important contributor to responses in the paw task. However, Essler et al. (2017) handed rewards to subjects in their study, whereas Brucks et al. (2017a) did not; the physical interaction resulting from handing rewards to the subjects may, therefore, be important in influencing responses in these tasks. It may be the case that the physical interaction contributes simply by directing the subjects' attention towards their partner's payoffs. In this context, it is important to mention that dogs in the paw task seem to fixate on the experimenter's hands and follow them persistently. It is, therefore, conceivable that the position of the experimenter's hands has a considerable influence over what the subjects attend to and perceive in the paw task. In fact, in the current study, subjects seemed to spend the longest duration gazing at their own bowl (though this was not significant) in the FC condition, which is also the condition in which the experimenter's hand enters the bowl most frequently (as it takes a piece of HVR, shows it to the subject, returns this piece to the bowl, and then takes a piece of LVR to give to the subject). Furthermore, subjects spent the shortest durations gazing at their own bowl in the RI and NR conditions in which the experimenter's hand does not enter the subject's bowl at all.

It is important to note that although subjects were not inequity averse in Brucks et al.'s (2017a) buzzer task, they did give up in the RI condition. The conclusion that they were not inequity averse is due to the lack of a difference in performance between the RI and NR conditions. However, subjects' level of performance in the RI condition in both versions of the buzzer task (experimenter present and experimenter absent) was similar to typical performances in the RI condition of the paw task, including the current study. It is possible that the level of motivation to perform the buzzer task without reward was too low to allow inequity aversion to emerge. An alternative possibility, however, is that the motivation to work in the absence of reward was exaggerated in the NR condition of those studies that found inequity aversion. For example, the control movement of the food in the experimenter's hand without any clear aim or recipient may have created a false expectation of reward attainability. This may have encouraged the dogs to work longer than they might have, otherwise. Consequently, dogs' refusals to continue working in the RI condition of inequity tasks to date may have been based on perceived attainability of rewards rather than social comparison.

In conclusion, this study indicates that a shared food source is not necessary to elicit inequity aversion in dogs and the presence of separate food sources for each dog is unlikely to explain the lack of inequity aversion in the buzzer task carried out by Brucks et al. (2017a). Future studies should explore other factors that may be important in influencing inequity aversion, such as the presence of a shared experimenter, while other potential explanations for the results of Brucks et al. (2017a) should also be investigated in an attempt to further our understanding of factors that influence inequity aversion in dogs.

## Author Contributions

JM and FR designed the experiment. SA, IL, and CA conducted the experiment and coded the videos. JM analysed the data and wrote the manuscript. FR contributed to the analyses and to the writing of the manuscript.

### Conflict of Interest Statement

The authors declare that the research was conducted in the absence of any commercial or financial relationships that could be construed as a potential conflict of interest.
